# The association between disability and mortality among children and adolescents: A systematic review and meta-analysis

**DOI:** 10.1371/journal.pmed.1004947

**Published:** 2026-09-30

**Authors:** Maureen Moyo-Chilufya, Arthemon Nguweneza, Sara Rotenberg, Hannah Kuper, Tracey Smythe

**Affiliations:** 1 Department of Health and Rehabilitation Sciences, Division of Physiotherapy, Stellenbosch University, Cape Town, South Africa; 2 Department of Population Health, International Centre for Evidence in Disability, London School of Hygiene & Tropical Medicine, London, United Kingdom; 3 Missing Billion Initiative, Seattle, Washington, United States of America; London School of Hygiene and Tropical Medicine, UNITED KINGDOM OF GREAT BRITAIN AND NORTHERN IRELAND

## Abstract

**Background:**

Mortality is a fundamental marker of population health and equity. Despite major global declines in mortality over recent decades, disparities in survival persist across childhood and adolescence, particularly by disability status. We aimed to synthesise current evidence on all-cause mortality among children and adolescents with disabilities compared to their non-disabled peers through a systematic review and meta-analysis.

**Methods and findings:**

This review was prospectively registered with PROSPERO (CRD420251072039). We searched Embase, Global Health, MEDLINE, PubMed and PsycInfo to identify relevant studies published from January 1, 2000 to August 20, 2026. We included cohort studies that compared all-cause mortality rates for children and adolescents with and without disabilities (aged 0–19 years). We excluded studies without a clear definition of disability, disaggregated mortality data, or a control group. Two authors independently screened studies, with data extraction by one and verification by a second. We assessed risk of bias using a Joanna Briggs Institute critical appraisal tool. We conducted meta-analyses in RevMan for studies reporting age-sex-adjusted or multivariable-adjusted mortality ratios and R software to assess for publication bias. We reported heterogeneity using the *I*^*2*^ and *τ*^*2*^ statistics. We identified 12,664 records of which 29 studies met the inclusion criteria, representing over 35 million children/adolescents. Sixteen studies were eligible to be included in the meta-analysis. Overall, children with disabilities had a 5.31-fold (95% confidence interval (CI) [3.63,7.77]; *p* < 0.001; *τ*^*2*^ = 0.88; *I*^2^ = 98%) higher all-cause mortality than their peers. The association between disability and mortality was higher in both younger children 0–5 years (Hazard ratio (HR)=7.51: 95%CI [4.70,12.00]; *p* < 0.001; *τ*^*2*^ = 0.44; *I*^*2*^ = 91%) and children 6–19 years (HR = 4.68: 95%CI [2.26,9.69]; *p* < 0.001; *τ*^*2*^ = 0.93; *I*^*2*^ = 96%). The association varied by disability type and was most stark for children with neurological disabilities (HR = 11.71: 95%CI [7.90,17.35]; p < 0.001; *τ*^*2*^ = 0.19; *I*^*2*^ = 76%). Interpretation is limited by substantial between-study heterogeneity, differences in methods used to ascertain disability ascertainment and in the effect measures used, and the small number of studies from low-income and middle-income countries.

**Conclusions:**

Children with disabilities experience substantially higher mortality than their peers without disabilities. There is an urgent need for evidence on what works to close the mortality gap.

## Author summary

**Why was this study done?:**

- A systematic review published in 2024 reported a 4-fold higher mortality among children with disabilities in low- and middle-income countries, compared to their peers without disabilities, based on a meta-analysis of four studies.
- Mortality among children with disabilities has not previously been synthesised across all income settings globally, although individual studies indicate a higher mortality among children with disabilities.
- This study investigates the association between disability and all-cause mortality among children and adolescents and how this varies by age and impairment type.

**What did researchers do and find?:**

- We included 29 studies in the systematic review, of which 16 studies provided sufficient data for inclusion in the meta-analysis, contributing 26 comparative analyses.
- This study found a 5.31-fold higher (95% CI [3.63,7.77]; *p* < 0.001; *τ*^*2*^ = 0.88; *I*^*2*^ = 98%) mortality for children and adolescents with disabilities compared to those without.
- Children between 0 and 5 years had a 7.51-fold higher mortality, and those aged between six and 19 years had a 4.68-fold higher mortality compared with their peers without disabilities.

**What do these findings mean?:**

- This study demonstrates that children with disabilities experience substantial inequalities in mortality.
- There is limited evidence on the causes of these disparities and the proportion that may be avoidable, but given the diversity of impairments included in this study, the elevated mortality is unlikely to be due to impairment alone.
- Our review was limited by heterogeneity in disability definitions and analytical methods, and by the small number of studies from low-income and middle-income countries

## Abstract 3

In this systematic review and meta-analysis, Maureen Moyo-Chilufya and colleagues synthesize evidence from more than 35 million children and adolescents to examine differences in all-cause mortality by disability status. The study finds that children and adolescents with disabilities experienced higher mortality rates than their non-disabled peers, with variations across age groups and disability types, while highlighting important evidence gaps, particularly in low- and middle-income countries.

## Introduction

There are approximately 240 million children with disabilities globally, the majority of whom live in low and middle-income countries (LMICs) [1]. Disability refers to a broad range of impairments, which in interaction with environmental and personal factors, may affect a child’s functioning and participation in society on an equal basis with others [2]. Consequently, children and adolescents with disabilities represent an extremely diverse group that includes young people with different impairment types, genders and living in different parts of the world. Despite this diversity, children with disabilities experience commonalities such as stigma [3], societal exclusion [4,5], lower levels of education and higher levels of poverty [6]. Children with disabilities also have worse health outcomes than their peers without disabilities. For example, children with disabilities have a higher prevalence of malnutrition, including being stunted, wasted and underweight [7,8], as well as higher odds of asthma [9], and infectious diseases [10], compared to those without disabilities.

Children with disabilities may experience a higher risk of poor health due to several factors. First, adverse social determinants of health, such as limited inclusion in education, family poverty, negative societal attitudes, social isolation, exclusion from recreational or community activities [11,12], as well as violence and abuse [13], can adversely affect health. These exposures can affect a child’s health-related behaviours, and psychological and emotional development [14,15]. Second, the underlying impairment or condition may lead to adverse health outcomes. For example, children with Down syndrome are predisposed to certain cancers [16,17] and heart defects [18,19]. Third, health system failures, such as inaccessible health services and limited training around disability for child health professionals, can create barriers to accessing quality healthcare [20].

Recent reviews have identified a 2-fold elevated mortality rate among people with disabilities in LMICs [21], and globally [22]. However, the majority of existing literature has concentrated on adults with less focus on children and adolescents with disabilities. The review of data from LMICs only included 476 children with disabilities aged under 18 years who had died, and found mortality was four times higher among children with disabilities than their peers without disabilities. The global review did not disaggregate mortality by age. Other studies have reviewed mortality rates experienced by young people with specific impairments or conditions. For example, children with intellectual disabilities experience a 10 times higher rate of premature mortality compared to their peers without disabilities [23]. However, there is a lack of synthesised evidence on mortality among children with disabilities globally.

We aimed to quantify the association between disability and all-cause mortality among children and adolescents compared with those without disabilities, and to examine whether this association varied by age and disability category. This systematic review and meta-analysis therefore aimed to answer the following questions: what is the extent, strength, and quality of the published evidence of the association of disability and risk of all-cause mortality in children and adolescents; and to what degree does disability affect the risk of all-cause mortality, and does this risk vary based on age and impairment type?

## Methods

### Search strategy and selection criteria

This study is reported as per the Preferred Reporting Items for Systematic Reviews and Meta-Analyses (PRISMA) guideline (S1 Checklist). The protocol was registered *a priori* with the International Prospective Register of Systematic Reviews (PROSPERO: CRD420251072039). We searched Embase, Global Health, MEDLINE, PubMed and PsycInfo articles published from January 1, 2000 to August 20, 2026. The 2000 search start date was chosen to ensure that the included studies reflect associations between mortality and disability among children and adolescents that align with healthcare practices and diagnostic criteria. We included studies in any language that examined disability and mortality among children and adolescents globally (Table A in S1 Appendix).

### Study eligibility

Eligible articles included cohort and other longitudinal studies that assessed the association of disability and mortality (cause-specific or all-cause) among children and adolescents up to the age of 19 years from any global region.

Disability was conceptualised in accordance with the United Nations Convention on the Rights of Persons with Disabilities (2006) which states that *“Persons with disabilities include those who have long-term physical, mental, intellectual or sensory impairments which in interaction with various barriers may hinder their full and effective participation in society on an equal basis with others* [2].” We operationalised this definition by focussing on disability measured through limitations in functioning, activity limitations, or self-report (e.g., Washington Group on Disability Statistics questions, activities of daily living), or based on registration or certification (e.g., disability allowance recipient). Conditions such as spina bifida, cerebral palsy and specific impairments (e.g., visual, hearing, or physical impairments and intellectual and developmental disability) were included. No minimum duration of disability was required. Disability status could be determined through self-report, physician diagnosis, or standardised assessment tools.

The outcomes of interest were all-cause mortality or cause-specific mortality. Mortality could be reported using different measures of effect size, and we included all measures. We also included cause of death and death rate.

Studies were required to include a comparator group of people without disabilities or the general population (in which children with disabilities represent a subgroup of this population). Conference abstracts were considered during screening to maximise search sensitivity.

We excluded studies that did not have a clear definition of disability as per our working definition. We also excluded studies that did not have a comparator group of people without disabilities or did not report mortality data for children and adolescents separately from adults. Studies in which all participants had a systemic disease (e.g., diabetes) were not eligible for inclusion to support generalisability of findings. Grey literature was excluded.

### Data search and extraction

The electronic search and deduplication were conducted by TS, and the results were stored and screened using Covidence, and we used EndNote 2025 as the reference manager Study selection was conducted independently by TS and MMC. Each reviewer independently screened all the titles and abstracts against the eligibility criteria. From these preliminary lists, the reviewers independently assessed the full articles for inclusion, recording and discussing the reasons for exclusion at each stage.

We developed a standardised data-extraction form in Microsoft Excel. The data extraction form was piloted on five studies to refine the final version. One reviewer conducted the data extraction independently (MMC) and rechecked by a second reviewer (AN).

The outcome of interest was all-cause or cause-specific mortality for children and adolescents aged 0–19 years. The effect measures of interest included hazard ratios (HR), mortality rate ratios (MRR), odds ratios (OR), and standardised mortality ratios (SMR) with respective 95% confidence intervals (CI) overall, for age-specific groups and by disability category. We categorised disability according to the designation of authors as much as possible in alignment with disability-inclusive language. We calculated a hazard ratio where an effect measure was unavailable, and we reported the comparator analysis as determined by the authors where sufficient data was provided (numerator and denominator for each group). Data were summarised according to all-cause and cause-specific mortality.

### Risk of bias

Risk of bias assessment for all included studies was evaluated using the Joanna Briggs Institute (JBI) Critical Appraisal Checklist for Cohort Studies [24] to assess methodological quality and the extent to which each study addressed potential sources of bias in its design, conduct, and analysis. Studies were rated as low risk (if the total score was 10 or the maximum score of 11), medium risk (score of 8 or 9) and high risk (score of 7 or below).

One reviewer (MMC) conducted the initial quality assessment for each included study. All scores were independently verified by a second reviewer (AN). Any discrepancies in risk of bias ratings were resolved through discussion until consensus was reached.

### Statistical analysis

We used HRs with 95% CI to describe the summary results of meta-analyses. A HR more than 1 indicates higher mortality in children and adolescents with disabilities relative to the comparison group, whereas a HR less than 1 indicates lower mortality relative to the general population or control group [25]. The SMR were reported variably across studies and often expressed as percentages. We therefore converted index values to ratio measures by dividing by 100 to ensure comparability with other effect measures. We treated RRs and ORs as relative effect measures comparable to HRs under the rare-outcome assumption [25]. We analysed all effect estimates on the natural logarithmic scale using the generic inverse variance method with random effects. Standard errors were derived from published 95% CIs. For the pooled estimates, the 95% CIs were calculated by the Wald-type method and the *τ*^2^ was calculated by the DerSimonian and Laird method. The *Z*-test statistic was used to generate the p-values for the pooled estimates.

We selected age-sex adjusted estimates *a priori* for the meta-analysis where these were available. Multivariable-adjusted models adjusted inconsistently for socioeconomic status, comorbidities, and healthcare-related factors and may also have over-adjusted for variables on the causal pathway between disability and mortality. This approach has been adopted in previous studies [21,22]. When age- and sex-adjusted estimates were unavailable, we used multivariable-adjusted estimates in preference to unadjusted estimates. We excluded studies that only reported crude estimates from the meta-analysis. We assessed heterogeneity across studies using the *χ*^2^ test with Cochran’s *Q* statistic and quantified it using Higgins’ *I*^*2*^ statistic and *τ*-squared statistic.

We anticipated high heterogeneity between studies in the overall meta-analysis, and we therefore undertook subgroup analyses by age and disability category. We performed separate meta-analyses for the two age categories (≤5 years old and 6–19 years old) given the potential for effect modification by age. We undertook meta-analyses by different impairment types (intellectual, and developmental, multiple, neurological and, physical) where all-cause mortality data outcome measures were available for three or more analyses. We conducted sensitivity analyses restricted to one estimate per study. We also conducted meta-analyses separately for age- and sex-adjusted and multivariable-adjusted estimates. We examined funnel plot asymmetry and used Egger’s regression test to assess for publication bias.

All statistical analyses were performed using RevMan version 9.9.0 (Cochrane Collaboration) and R statistical software.

### Deviations from protocol

Initially, our registered PROSPERO inclusion criteria included children up to 18 years old. However, we identified several studies that classified adolescents as up to 19 years old during full-text review, and we therefore expanded the age range by one year to avoid excluding relevant data based on a narrow age cutoff.

We did not conduct any meta-analyses on the cause-specific outcomes and avoidable mortality due to limited data. Additionally, there was insufficient data to stratify by sex. We assessed the methodological quality of included studies using the Joanna Briggs Institute (JBI) critical appraisal tools, which we considered more appropriate than GRADE for evaluating risk of bias in this heterogeneous body of observational evidence.

## Results

### Study selection

We identified 12,664 titles to be screened, and following full-text review, identified 29 studies that met our inclusion criteria [23,26–53]. The PRISMA diagram (Fig 1) outlines the screening process and reasons for exclusion. The most common reason for exclusion was the lack of disaggregated mortality data for children and adolescents (n = 20; 38%). Detailed reasons for exclusion are provided (Table B in S1 Appendix).

**Fig 1:**
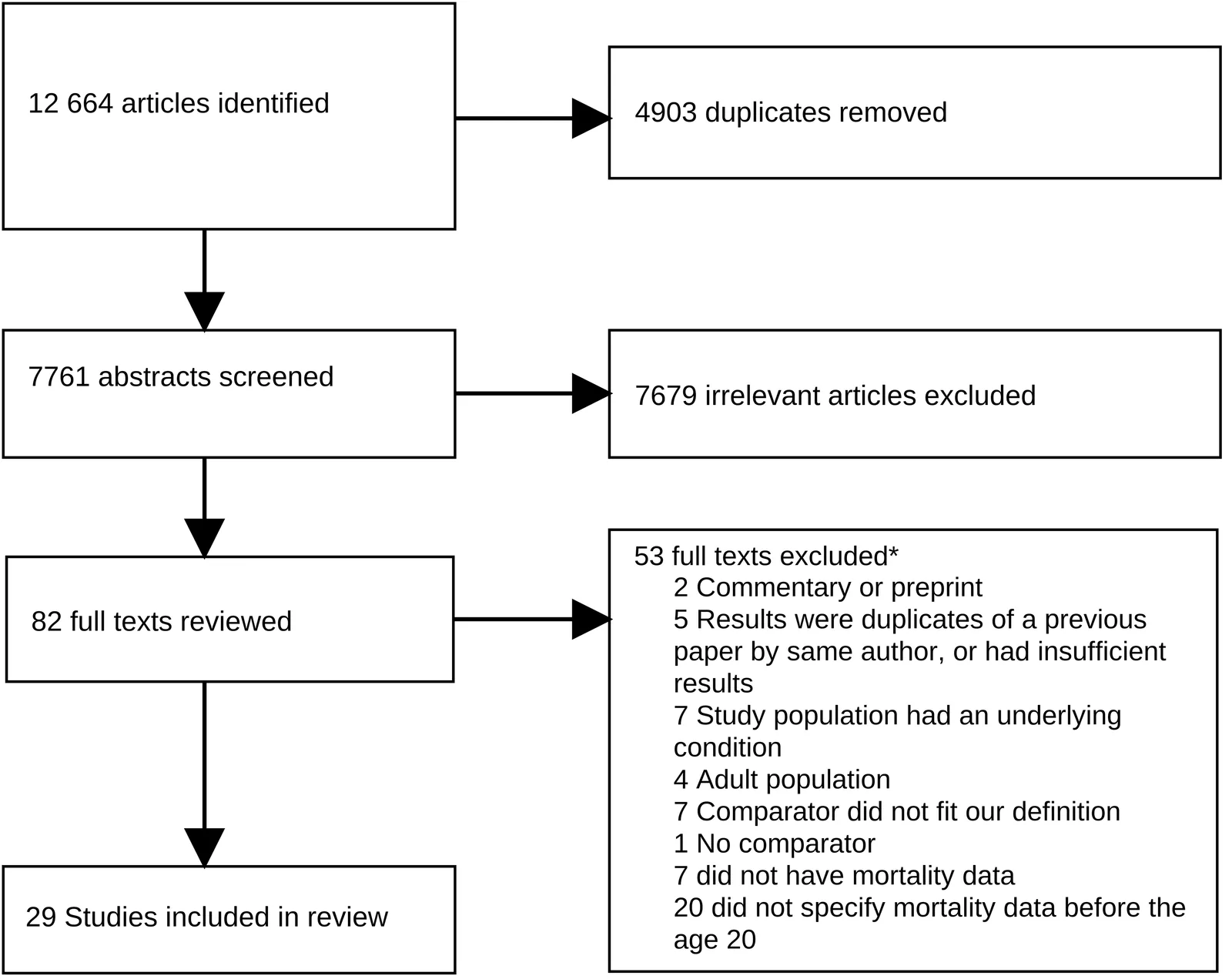
Selection of studies. *Reasons for full study text exclusion and titles provided in Table B in S1 Appendix.

### Study characteristics

All the included studies were prospective or retrospective cohort designs (Table 1). Notably, more than 35 million children and adolescents were included in these studies from 16 countries: Australia (*n* = 3), England (*n* = 5) Denmark (*n* = 3), Scotland (*n* = 4), South Korea (*n* = 3), USA (*n* = 2), while Brazil, Finland, Ireland, Kenya, Sweden, Taiwan, Uganda and New Zealand each presented one study. There was only one study from the United Kingdom representing England, Scotland, Wales and Northern Ireland. Four of WHO world regions were represented, excluding South-East Asia and the Eastern Mediterranean region. Additionally, nearly half of the studies were from Europe (*n* = 16; 55%). Most studies drew on national databases or registries (*n* = 21; 72%) and there was a marked increase in publications between the years 2020 and 2026 (*n* = 15; 52%). Sample size by age was not reported in over a third of the studies (*n* = 10; 34%). Among those that did, sizes were typically either less than or equal to 50,000 (*n* = 7; 24%) or more than 2,000,000 (*n* = 7; 24%). The most represented disability domain was intellectual and developmental disability (*n* = 17; 59%) and many of the disability assessments were by medical records (*n* = 21; 72%). There were only three studies that entirely focussed on the age group 0–5 years (*n* = 3; 10%). Sex-disaggregated data for children and adolescents by age, were unavailable in most of the studies (*n* = 17; 59%) and all included studies had a medium to low risk of bias (Table C in S1 Appendix). Seventeen studies (59%) included children with intellectual and developmental disabilities (comprised of Attention Deficit Hyperactivity Disorder, Autism and Down syndrome). Eight studies (28%) focussed on neurological impairments such as cerebral palsy and congenital Zika virus syndrome, and two studies (7%) with physical impairment (achondroplasia and osteogenesis imperfecta) and two studies (7%) included children with multiple impairments (Table D in S1 Appendix).

**Table 1:** Characteristics of studies (*n* = 29 Cohort studies).

Category	Characteristics of studies	*N* (%)
WHO Region	Western Pacific Region	8 (28%)
	Region of the Americas	3 (10%)
	African region	2 (7%)
	European Region	16 (55%)
	South-East Asia Region	0 (0%)
	Eastern Mediterranean Region	0 (0%)
Data source	Community based cohorts	6 (21%)
	Hospital or clinic	2 (7%)
	Databases/registries	21 (72%)
Five-year range of publication	2000–2004	3 (10%)
	2005–2009	1 (3%)
	2010–2014	1 (3%)
	2015–2019	9 (31%)
	2020–2026*	15 (52%)
Sample size	≤50,000	7 (24%)
	>50,000 ≤1,000,000	2 (7%)
	>1,000,000 ≤ 2,000,000	3 (10%)
	>2,000,000	7 (24%)
	Not reported**	10 (34%)
Population disability domain	Multiple impairments***	2 (7%)
	Intellectual & developmental disability	17 (59%)
	Neurological impairment	8 (28%)
	Physical impairment	2 (7%)
Age group	0–5 years	3 (10%)
	6–19 years	26 (90%)
Sex (% Girls)	0%–50%	11 (38%)
	5%1–100%	1 (3%)
	Not reported	17 (59%)
Disability assessment	Assessed by a clinician	1 (3%)
	Medical records	21 (72%)
	Both clinician assessment and medical records	7 (24%)
Death data source****	Verbal autopsy or participant follow-up	2 (7%)
	Population registers or national death record	22 (76%)
	Death certificate	1 (3%)
	Hospital records	4 (14%)
Risk of bias (JBI Critical Appraisal)	Low risk (score = 10–11)	27 (93%)
	Medium risk (score = 8–9)	2 (7%)
	High risk (score ≤7)	0 (0%)

*The years of publication are presented in five-year intervals except the last category which includes 2026.

**Some studies reported a general sample size but did not provide a disaggregated figure for children/adolescents and we have described these as “Not reported”.

***The results are presented per study (*n* = 29). Where studies had results for more than one disability type, they are classified in the multiple impairments domain. These studies included hearing and visual impairment.

****The data source refers to the place where the participant and mortality data were collected from.

The 29 studies reported associations between disability and mortality across 63 separate analyses groups. Of these, 46 (73%) analyses compared all-cause mortality (Table 2), while 17 (27%) analyses compared cause-specific mortality (Table 3), in children with and without disabilities and were eligible for potential inclusion in the meta-analysis.

**Table 2:** Association of disability and all-cause mortality (*n* = 46 comparisons).

Author (Year)	Country	Age category	With disability	Without disability	Deaths disability	Deaths Controls	Measure of effect	Unadjusted effect estimate (95% CI)	Age-sex adjusted effect estimate (95% CI)	Multivariable adjusted effect estimate (95% CI)
Hearing Impairment										
Decoufle (2002)	USA	10–19 years	–	–	3	–	MR	5.0 (1.0,14.5)	–	–
Intellectual and developmental disability										
Bourke (2017)	Australia	1–5 years	–	–	–	–	HR	9.5 (7.8,11.5)	6.0 (4.8,7.6)	–
Bourke (2017)	Australia	6–10 years	–	–	–	–	HR	15.9 (11.9,21.2)	12.6 (9.0,17.7)	–
Decoufle (2002)	USA	10–19 years	–	–	23	–	MR	3.3 (2.1,5.0)	NR	–
Huang (2021)	Taiwan	6–17 years	2,173	21,730	11	55	OR	–	2.04 (1.05,3.93)	–
Hughes-McCormack (2022)	Scotland	5–9 years	1,435	34,607	–	–	SMR	NA	27.4 (20.6,36.3)	–
Hughes-McCormack (2022)	Scotland	10–14 years	1,879	37,514	–	–	SMR	NA	15.8 (12.7,19.8)	–
Hughes-McCormack (2022)	Scotland	15–19 years	2,063	40,990	–	–	SMR	NA	8.6 (6.9,10.7)	–
McCarron (2015)	Ireland	0–19 years	11,709	1,275,078	272	5,006	SMR	NA	668 (591,752)	–
Smith (2020)	Scotland	5–14 years	>28,297	>1,332,123	NR	NR	SMR	NA	21.6 (16.6,28.2)	–
Dalsgaard (2015)	Denmark	1–5 years	–	–	10	1,184	MRR	2.23 (1.11–3.91)	1.97 (0.99,3.46)	1.86 (0.93,3.27)
Dalsgaard (2015)	Denmark	6–17 years	–	–	59	1,829	MRR	1.83 (1.40–2.35)	1.63 (1.25,2.09)	1.58 (1.21,2.03)
Hughes-McCormack (2020)	Scotland	1–12 months	–	–	53	12	RR	22.8 (17.5, 32.9)	–	–
Kim (2021)	South Korea	6–13 years	32,878	3,566,026	237	9,843	HR	2.6 (2.3,3.0)	–	2.5 (2.2,2.9)*
Sherrard (2001)	Australia	8–19 years	–	–	5	–	SMR	NA	8 (–)	–
Shumway (2025)	England	0–16 years	10,621	9,621,025	–	–	HR	15.3 (14.2,16.5)	–	–
Smith (2021)	Scotland	0–19 years	9,754	777,912	6	458	SMR	NA	1.1 (0.5,2.5)	–
Sun (2019)	Sweden	1–17 years	–	–	40	2,435	HR	1.58 (1.09–2.29)	1.41 (0.97,2.04)	–
Tibby (2012)	England	0–16 years	1,278	32,207	54	1,997	SMR	NA	0.83 (0.63,1.09)	–
Yoo (2022)	South Korea	0–8 years	35,529	4,953,822	–	–	HR	2.34 (2.06,2.65)	–	–
Multiple disability domain										
Decoufle (2002)	USA	10–19 years	–	–	19	–	MR	5.8 (3.5,9.1)	–	–
Bowden (2026)	New Zealand	0–19 years	–	–	–	–	RR	–	4.83 (3.43,6.00)	–
Neurological disability										
Abuga (2019)	Kenya	6–9 years	306	22,873	11	272	SMR	3.13 (1.7,5.7)	3.15 (1.66,5.49)	–
Decoufle (2002)	USA	10–19 years	–	–	14	–	MR	11.4 (6.2,19.1)	–	–
Gimeno (2026)	England	4–11 years	51,289	2,300,300	466	695	HR	30.07 (20.28,44.59)	–	–
Hinwood (2023)	Australia	<1 year	–	–	36	–	MR	7.5 (5.06,9.97)	–	–
Hinwood (2023)	Australia	1–4 years	–	–	123	–	MR	6.5 (5.38,7.69)	–	–
Hinwood (2023)	Australia	5–9 years	–	–	113	–	MR	5.3 (4.32,6.27)	–	–
Hinwood (2023)	Australia	10–14 years	–	–	93	–	MR	5.2 (4.14,6.25)	–	–
Hinwood (2023)	Australia	15–19 years	–	–	95	–	MR	6.6 (5.26,7.90)	–	–
Kim (2019)	South Korea	18–24 months	–	–	–	–	HR	–	11.64 (6.32,21.44)	–
Kim (2019)	South Korea	30–36 months	–	–	–	–	HR	–	11.27 (5.92,21.46)	–
Kim (2019)	South Korea	42–48 months	–	–	–	–	HR	–	15.38 (8.44,28.02)	–
Kim (2019)	South Korea	54–60 months	–	–	–	–	HR	–	18.03 (9.13,35.62)	–
Kim (2019)	South Korea	66–71 months	–	–	–	–	HR	–	24.85 (9.97,61.91)	–
Macaulay (2026)	England	11–12 years	–	–	–	–	HR	27.33 (21.90,33.99)	–	–
Namaganda (2020)	Uganda	2–5.9 years	–	–	8	93	MRR	21.0 (10.2,43.2)	–	–
Namaganda (2020)	Uganda	6–9.9 years	–	–	3	31	MRR	27.0 (8.2,88.2)	–	–
Namaganda (2020)	Uganda	10–17.9 years	–	–	4	45	MRR	39.6 (14.2,110.0)	–	–
Cardim (2025)	Brazil	0–5 years	3,080	11,384,351	444	114,838	HR	14.66 (13.35,16.09)	–	13.1 (11.86,14.5)**
Zylbersztejn (2025)	England	0–10 years	127,534	3,452,691	7,769	11,274	RR	18.66 (18.1,19.2)	–	–
Physical Impairments										
Folkestad (2016)	Denmark	0-19 years	687	3 435	26	–	HR	66.1 (15.7,278.7)	–	68.1 (16.2,287.3)***
Hashmi (2018)	USA	<1 year	–	–	1	–	SMR	Not applicable	13.8 (1.9,97.8)	–
Hashmi (2018)	USA	1–4 years	–	–	5	–	SMR	Not applicable	239.8 (99.8,576)	–
Hashmi (2018)	USA	5–14 years	–	–	2	–	SMR	Not applicable	29.9 (7.5,119.6)	–
Visual disability										
Decoufle (2002)	USA	10–19 years	–	–	6	–	MR	17.1 (16.3, 37.2)	–	–

*Multivariable adjusted effect estimate for birth year and household income.

**Adjusted effect estimate for region, year of birth, maternal age, maternal education, maternal race or ethnicity and marital status.

***Adjusted effect estimate for comorbidities.

Abbreviations: HR, Hazard ratio; MR, Mortality ratio; OR, Odds ratio; RR, Rate ratio; SMR, Standardised mortality ratio; USA, United States of America; NA, Not applicable; –, Not reported.

**Table 3:** Association of disability and cause-specific mortality (*n* = 17 comparisons).

Author (Year)	Country	Impairment category	Cause of mortality	Age Category	Deaths disability (*n*)	Deaths Controls (*n*)	Measures of Effect	Unadjusted effect estimate (95% CI)	Age-sex adjusted effect estimate (95% CI)	Multivariable adjusted effect estimate (95% CI)
Patja (2001)	Finland	Intellectual and developmental disability	Infectious diseases	2–19 years	2	–	RR	–	3.2 (0.0,3.2)	–
Patja (2001)	Finland	Intellectual and developmental disability	Tumours	2–19 years	0	–	RR	–	0.0 (0.0,0.1)	–
Patja (2001)	Finland	Intellectual and developmental disability	Vascular diseases	2–19 years	0	–	RR	–	0.0 (0.0,0.1)	–
Patja (2001)	Finland	Intellectual and developmental disability	Respiratory diseases	2–19 years	6	–	RR	–	4.3 (0.3,4.7)	–
Patja (2001)	Finland	Intellectual and developmental disability	Diseases of the digestive system	2–19 years	1	–	RR	–	0.8 (0.0,4.1)	–
Patja (2001)	Finland	Intellectual and developmental disability	Accidents and poisoning	2–19 years	1	–	RR	–	0.1 (0.0,0.2)	–
Patja (2001)	Finland	Intellectual and developmental disability	Other causes	2–19 years	9	–	RR	–	1.3 (0.2,1.4)	–
Sherrard (2001)	Australia	Intellectual and developmental disability	Injury-related	8–19 years	5	–	SMR	NA	8 (–)	–
Fitzgerald (2019)	Denmark	Intellectual and developmental disability	Suicide	10–19 years	7	2,197	IRR	0.35 (0.17,0.74)	–	–
McCarthy (2009)	England, Scotland, Wales and Northern Ireland	Intellectual and developmental disability	Suicide	11–14 years	2	56	SMR	NA	161.91 (19.61,584.88)	–
Decoufle (2002)	USA	Multiple domains	Nervous system	10–19 years	6	–	MR	17.4 [6.4, 37.9]	–	–
Decoufle (2002)	USA	Multiple domains	Cardiovascular system	10–19 years	5	–	MR	12.3 [4.0,28.7]	–	–
Decoufle (2002)	USA	Multiple domains	Injuries/ poisoning	10–19 years	6	–	MR	0.8 [0.3, 1.8]	–	–
Decoufle (2002)	USA	Multiple domains	Other causes	10–19 years	13	–	MR	7.7 [4.1,13.2]	–	–
Cardim (2025)	Brazil	Neurological impairment	Nervous system disease	0–5 years	82	4,844	HR	64.0(51.5,79.6)	–	57.1(45.2,72.1)
Cardim (2025)	Brazil	Neurological impairment	Infectious and parasitic disease	0–5 years	152	17,948	HR	32.7 (27.9,38.4)	–	28.3(23.9,33.5)
Cardim (2025)	Brazil	Neurological impairment	Respiratory system disease	0–5 years	154	17,254	HR	34.5 (29.4,40.4)	–	30.3(25.6,35.8)

HR, Hazard ratio; IRR, Incidence rate ratio; MR, Mortality ratio; USA, United States of America; NA, Not applicable; –, Not reported; RR, Relative risk; SMR, Standardised mortality ratio.

### Meta-analysis

Twenty-six analyses from 16 studies were eligible to be included in the overall meta-analysis of childhood mortality (Fig 2). Children and adolescents with disabilities had a 5.31-fold (95% CI [3.63,7.77]; p < 0.001; *τ*^*2*^ = 0.88; *I*^*2*^ = 98%) higher all-cause mortality compared to their peers without disabilities. However, heterogeneity was very high, suggesting substantial variability between the studies.

**Fig 2:**
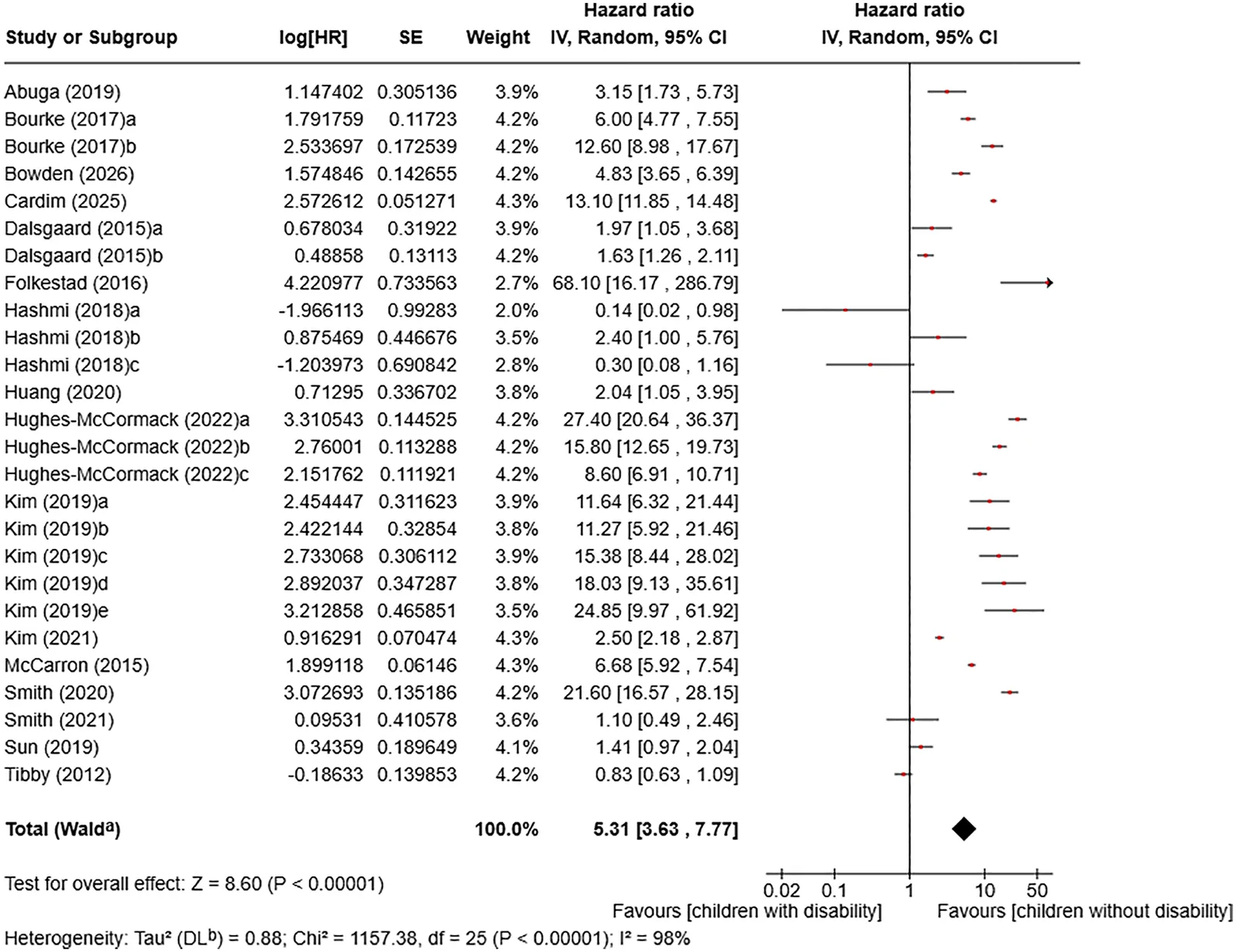
Association of disability and all-cause mortality among children and adolescents.

Children 0–5 years had a 7.51-fold (95%CI [4.70,12.00]; p < 0.001; *τ*^*2*^ = 0.44; *I*^*2*^ = 91%) higher all-cause mortality while those aged 6–19 years had a 4.68-fold (95%CI [2.26,9.69]; *p* < 0.001; *τ*^*2*^ = 0.93; *I*^*2*^ = 96%) higher all-cause mortality compared to their peers without disabilities. The heterogeneity for these analyses remained high (Fig 3).

**Fig 3:**
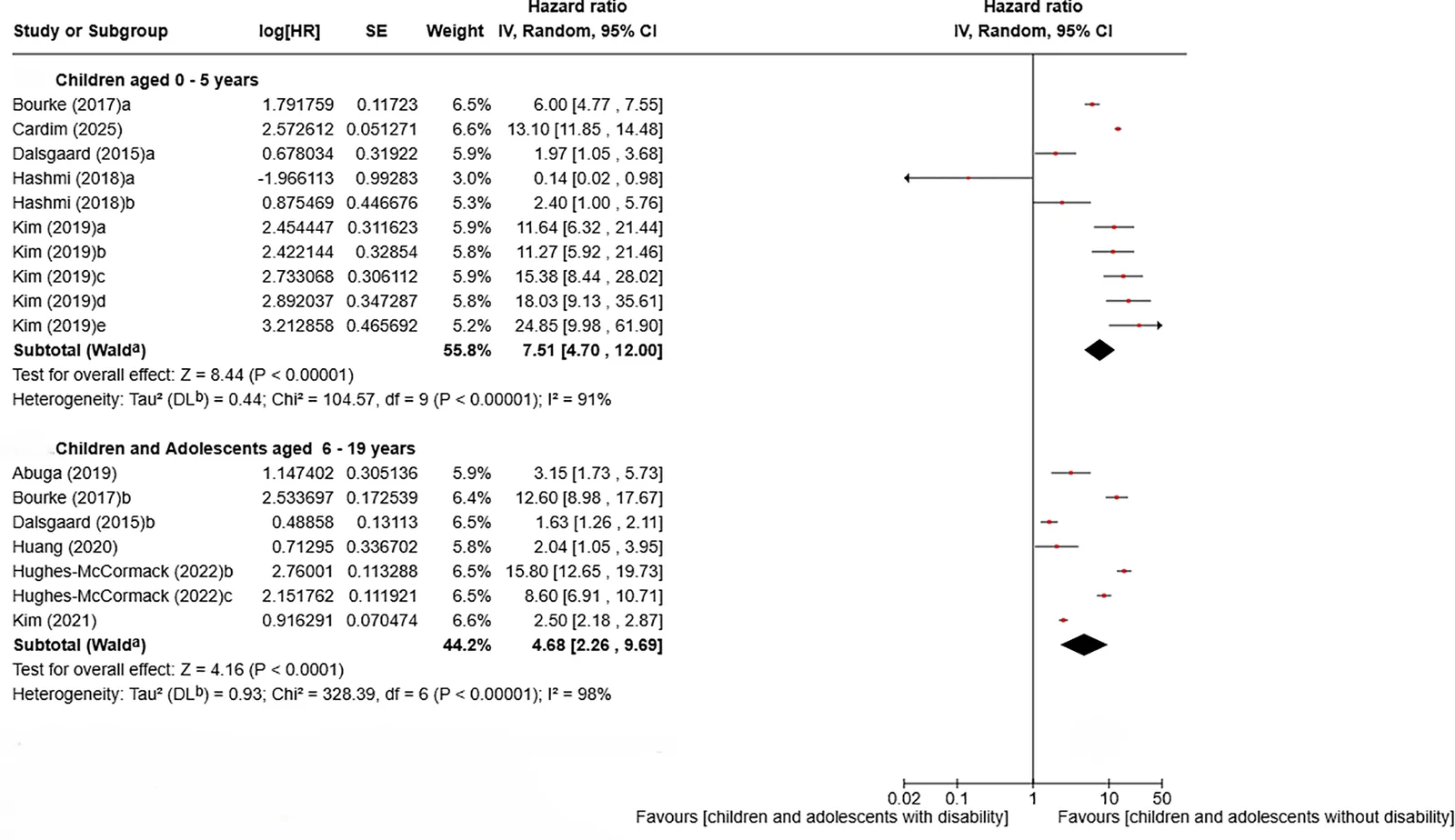
Association of disability and all-cause mortality among children aged 0–5 years and 6–19 years.

Children with neurological disabilities had an 11.71-fold (95%CI [7.90,17.35]; *p* < 0.001; *τ*^*2*^ = 0.19; *I*^*2*^ = 76%) higher all-cause mortality, and those with intellectual and developmental disabilities had a 4.45-fold (95%CI [2.63,7.53]; p < 0.001; *τ*^*2*^ = 0.97; *I*^*2*^ = 98%) higher mortality compared to their peers without disabilities. In contrast, children with physical impairments (HR = 1.69: 95%CI [0.16,18.13]; *p* = 0.660; *τ*^*2*^ = 5.32; *I*^*2*^ = 92%) showed no evidence of a difference in mortality compared to those without disabilities. Heterogeneity remained high for disability categories as shown in Fig 4. (Tables E–J in S1 Appendix Meta-analysis Table).

**Fig 4:**
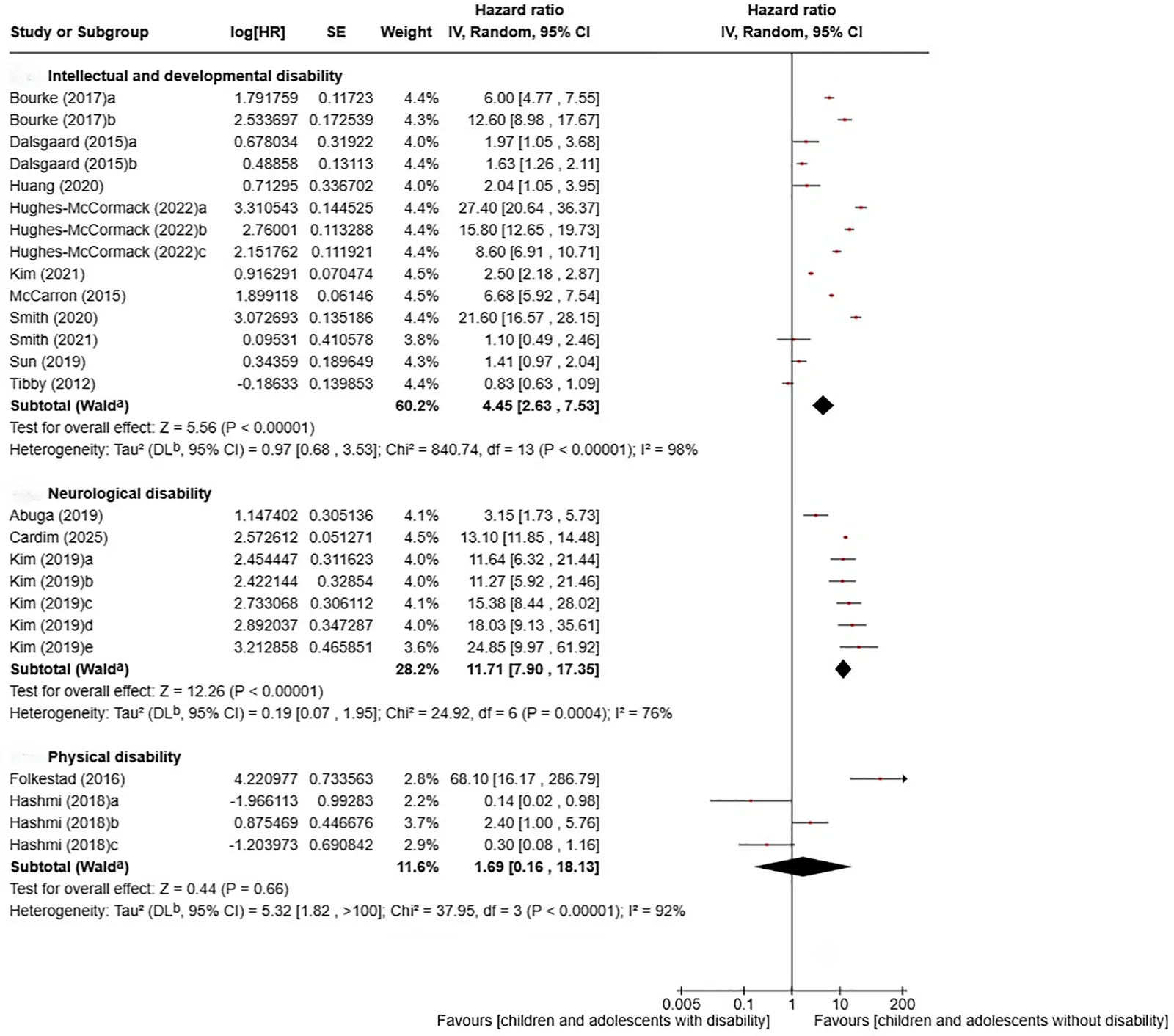
Association of disability type with all-cause mortality for children and adolescents.

The sensitivity analyses restricted to one estimate per study showed that the pooled effect size and direction of association remained consistent (Figs A–C in S1 Appendix). Disability remained strongly associated with mortality irrespective of adjustment strategy (Figs D and E in S1 Appendix).

It was not possible to conduct the meta-analysis for cause-specific mortality which were from six countries: USA (*n* = 1), Finland (*n* = 1), Brazil (*n* = 1), Denmark (*n* = 1) Australia (*n* = 1) and the United Kingdom (*n* = 1). Some of the notable specific causes of death among children with disability, as described by authors included infectious diseases, respiratory diseases, suicide, nervous system diseases and injuries and poisoning. (Table 3).

Visual inspection of the funnel plot suggested some asymmetry. However, Egger’s regression test did not provide statistically significant evidence of funnel-plot asymmetry (*p* = 0.073). There is no clear evidence of small-study effects or publication bias. (Fig F in S1 Appendix).

## Discussion

This systematic review and meta-analysis shows that children and adolescents with disabilities experience a 5.31-fold higher all-cause mortality compared to their peers without disabilities. Children aged 0–5 years had a 7.51-fold higher mortality, and those aged 6–19 years had a 4.68-fold higher mortality compared with their peers without disabilities. Elevated mortality varied by disability type. Children with neurological disabilities experience an 11.7-fold higher all-cause mortality, while those with intellectual and developmental disabilities have a 4.45-fold higher all-cause mortality among all age groups. We found no significant difference in all-cause mortality between children with and without physical impairments, although the small number of comparison groups in this category may have limited the precision of this estimate. We identified evidence gaps, including insufficient data to compare mortality by sex, geographic region, or several functional domains such as hearing or vision.

Our findings are in line with a previous meta-analysis from LMICs [21], which reported an all-cause mortality of 4.46(95%CI [3.01,6.59]) across four studies in children under 18 years with disabilities. The results also align with the wider evidence base showing higher mortality rates and poorer health outcomes among people with disabilities, including children [1], and wide health inequities for children with disabilities compared to those without [54,55].

Our review has several important strengths. To our knowledge, this is among the few quantitative synthesis of relative mortality among children and adolescents with disabilities globally. The review adhered to PRISMA standards and a prospectively registered PROSPERO protocol, with independent dual screening, data extraction, and risk-of-bias assessment using a validated tool to ensure methodological rigour and transparency. Standardised effect-size conversion and subgroup analyses by age and disability type strengthened the validity of pooled estimates. However, our study had some limitations that should be considered while interpreting the findings. The evidence base was concentrated in high-income settings and relied predominantly on administrative or medical records, limiting generalisability to LMICs and to children whose disabilities are not clinically identified or recorded. Few studies were available for several functional domains, including hearing and vision impairment, limiting impairment-specific analyses. We also identified substantial heterogeneity across studies, including differences in disability classification, age structure, mortality ascertainment, and adjustment strategies. We undertook the meta-analysis despite the heterogeneity, although we attempted to address this issue by conducting separate pooled analyses (e.g., age group, impairment type). Several additional limitations should be considered when interpreting the pooled analyses. We did not have sufficient data to analyse cause-specific mortality, or to conduct analyses by sex, disability severity or world region. Most studies reported broad age categories, which limited more detailed analyses across developmental stages. Some studies contributed to multiple age-stratified estimates although the sensitivity analyses indicated that our pooled estimates remained consistent. Further, as most of our studies used minimally adjusted estimates, residual confounding by social determinants of health cannot be excluded.

The observed higher mortality could be understood through three pathways. First, children with disabilities are more likely to experience adverse social determinants of health [56,57]. They are disproportionately affected by poverty, limited access to education, inadequate housing and social exclusion [54]. Second, the underlying impairment or health conditions may amplify risks to ill health, and constrain opportunities for wellbeing, as described in an umbrella review among people with autism [58]. Third, children with disabilities experience barriers to accessing healthcare, such as negative provider attitudes, insufficient training and skills for healthcare workers, transport difficulties, and inaccessible infrastructure [57,59].

These pathways likely interact to produce inequities in survival. For instance, children with disabilities have higher prevalence of malnutrition [7], which is associated with mortality. Although some impairment-related complications may contribute directly to mortality risk [23], evidence from cause-specific mortality studies suggests that many deaths occur from common, preventable conditions such as pneumonia [60]. Together, these findings are consistent with broader evidence showing inequities in access to prevention, timely diagnosis, and quality healthcare among children with disabilities [6]. Our review did not evaluate interventions directly, and the extent to which these mortality disparities are preventable remains uncertain. However, the UK Confidential inquiry into Premature Deaths of People with intellectual disabilities identified high proportions of avoidable deaths associated with delayed diagnosis, barriers to care, and inequities in the quality of health services received [60]. Evidence on effective interventions to reduce mortality among children with disabilities remains limited.

There are core ways in which health systems can improve to better meet the needs of children and adolescents with disabilities. First, disability needs to be embedded within routine health policy, planning, and financing to ensure that services are designed from the outset to reach children with diverse impairments [61,62]. Second, primary healthcare must be strengthened to provide early identification, referral, and family-centred support, with clear pathways between community services, rehabilitation, and specialised paediatric care [63]. Third, health systems must invest in accessible facilities, assistive technology, and communication supports so that children with disabilities can use services on an equal basis with others [62,64,65].

This review highlights evidence gaps and priorities for future research. Future studies should prioritise disability-inclusive longitudinal studies that use standardised measures of disability (e.g., Washington Group disability measurement) [66,67]. Linking disability data with mortality surveillance systems, administrative health databases, and birth or hospital records could improve understanding of mortality patterns across childhood and adolescence [23]. More evidence is also needed from LMICs, where disability-related mortality data remain particularly sparse. These countries could consider including disability identifiers within demographic surveillance systems, health information systems, and mortality databases, which may improve disability-disaggregated mortality monitoring [68]. Future studies should investigate avoidable mortality, causes of death, and barriers to accessing timely and appropriate healthcare among children and adolescents with disabilities. Future research should also collect data that supports robust modelling of age as a continuous variable.

Children with disabilities experience substantially higher mortality rates than their peers without disabilities throughout childhood and across different disability types. There is need for greater inclusion of children with disabilities in health systems, alongside research to identify effective approaches for reducing mortality and improving survival.

## Supporting information

S1 Checklist PRISMA 2020 Checklist. Page MJ, McKenzie JE, Bossuyt PM, Boutron I, Hoffmann TC, Mulrow CD, et al. The PRISMA 2020 statement: an updated guideline for reporting systematic reviews. https://www.bmj.com/content/372/bmj.n71. This work is licensed under CC BY 4.0. To view a copy of this license, visit https://creativecommons.org/licenses/by/4.0/. For more information please see: https://www.prisma-statement.org/citing-prisma-2020 – PRISMA.
(DOCX)

S1 Appendix Supplementary figures and tables. (DOCX)

## Abbreviations

CI: confidence interval
HR: Hazard ratio
LMICs: low and middle-income countries
MRR: mortality rate ratios
OR: odds ratios
PRISMA: Preferred Reporting Items for Systematic Reviews and Meta-Analyses
SMR: standardised mortality ratios
