# Supplementary material for: The association between disability and mortality among children and adolescents: A systematic review and meta-analysis
Source: PLoS Med. 2026 Sep 30;23(9):e1004947. doi: 10.1371/journal.pmed.1004947 (PMC13641705; doi:10.1371/journal.pmed.1004947)
Supplement: Supplementary file 2 [file pmed.1004947.s002.docx]

**S2 Appendix**

Contents

[Table A: Example of search terms	2](#_Toc240901640)

[Table B: Excluded full texts and reasons for exclusion(n=53)	5](#_Toc240901641)

[Table C: JBI Critical appraisal for cohort studies	7](#_Toc240901642)

[Table D: Disability definitions as reported by authors of individual studies (n=29 studies)	8](#_Toc240901643)

[Table E: Meta-analysis tables – Overall results (n=26 comparison groups)	10](#_Toc240901644)

[Table F: Results by age group (0 – 5 years) (n =10 comparison groups)	10](#_Toc240901645)

[Table G: Results by age group (6 – 19 years) (n =7 comparison groups)	10](#_Toc240901646)

[Table H: Intellectual and developmental disability (n =14 comparison groups)	10](#_Toc240901647)

[Table I: Neurological disability (n = 7 comparison groups)	11](#_Toc240901648)

[Table J: Physical disability (n = 4 comparison groups)	11](#_Toc240901649)

[Fig A: Sensitivity analysis for the overall association of disability and all-cause mortality among children and adolescents, restricted to one estimate per study	12](#_Toc240901650)

[Fig B: Sensitivity analysis for the association of disability and all-cause mortality among children aged 0 - 5 years and 6-19 years, restricted to one estimate per study	13](#_Toc240901651)

[Fig C: Sensitivity analysis for the association of disability type with all-cause mortality for children and adolescents, restricted to one estimate per study	14](#_Toc240901652)

[Fig D: Sensitivity analysis for the overall association of disability and mortality with only minimally adjusted effect sizes	15](#_Toc240901653)

[Fig E: Sensitivity analysis for the overall association of disability and mortality with only multivariable adjusted effect sizes	16](#_Toc240901654)

[Fig F: Funnel plot assessing publication bias in studies examining the association between disability and all-cause mortality among children and adolescents	17](#_Toc240901655)

## Table A: Example of search terms

| Disability | Searches |
| --- | --- |
| 1 | exp Disabled Child/ |
| 2 | ((child* or youth or adolescent* or girl* or boy* or teen* or juvenile* or infant* or baby* or toddler* or minor) adj1 disab*).ti,ab. |
| 3 | ((child* or youth or adolescent* or girl* or boy* or teen* or juvenile* or infant* or baby* or toddler* or minor) adj1 handicap*).ti,ab. |
| 4 | ((child* or youth or adolescent* or girl* or boy* or teen* or juvenile* or infant* or baby* or toddler* or minor) adj1 impair*).ti,ab. |
| 5 | ((child* or youth or adolescent* or girl* or boy* or teen* or juvenile* or infant* or baby* or toddler*) adj1 special need*).ti,ab. |
| 6 | 1 or 2 or 3 or 4 or 5 |
| 7 | (physical* adj1 (disab* or handicap* or impair* or abnormal* or defect*)).ti,ab. |
| 8 | ((congenital or genetic ) adj1 (disab* or handicap* or impair* or abnormal* or defect*)).ti,ab. |
| 9 | Cerebral Palsy/ |
| 10 | cerebral palsy.ti,ab. |
| 11 | Spinal Dysraphism/ |
| 12 | spinal dysraphism*.ti,ab. |
| 13 | spina bifida.ti,ab. |
| 14 | Muscular Disorders, Atrophic/ |
| 15 | (muscular adj1 (abnormal* or disorder* or condition* or disab* or impair* or dystroph*)).ti,ab. |
| 16 | Osteogenesis Imperfecta/ |
| 17 | Osteogenesis Imperfecta.ti,ab. |
| 18 | Musculoskeletal Abnormalities/ |
| 19 | (musculoskeletal adj1 (abnormal* or disorder* or condition* or disab* or impair*)).ti,ab. |
| 20 | amput*.ti,ab. |
| 21 | Clubfoot/ |
| 22 | clubfoot.ti,ab. |
| 23 | Poliomyelitis/ |
| 24 | polio*.ti,ab. |
| 25 | Paralysis/ |
| 26 | paraly*.ti,ab. |
| 27 | Paraplegi*.ti,ab. |
| 28 | quadriplegi*.ti,ab. |
| 29 | Hemiplegi*.ti,ab. |
| 30 | (spin* adj1 injur*).ti,ab. |
| 31 | wheelchair user*.ti,ab. |
| 32 | Albinism/ |
| 33 | albin*.ti,ab. |
| 34 | Dwarfism/ |
| 35 | Achondroplasia.ti,ab. |
| 36 | dwarf*.ti,ab. |
| 37 | Microcephaly.ti,ab. |
| 38 | hearing loss/ |
| 39 | hearing disorders/ |
| 40 | (hearing adj1 (loss or disab* or handicap* or impair* or abnormal* or defect*)).ti,ab. |
| 41 | (hard adj2 hearing).ti,ab. |
| 42 | deaf*.ti,ab. |
| 43 | Vision Disorders/ |
| 44 | (vision adj1 (disab* or handicap* or impair* or abnormal* or defect*)).ti,ab. |
| 45 | (visual* adj1 (disab* or handicap* or impair* or abnormal* or defect*)).ti,ab. |
| 46 | partial* sight*.ti,ab. |
| 47 | Intellectual Disability/ |
| 48 | (intellectual* or cognitive adj1 (disab* or handicap* or impair* or abnormal* or defect*)).ti,ab. |
| 49 | learning disabilities/ |
| 50 | (learning adj1 (disab* or handicap* or impair* or abnormal* or defect*)).ti,ab. |
| 51 | Developmental Disabilities/ |
| 52 | (development* adj1 (disab* or handicap* or impair* or abnormal* or defect*)).ti,ab. |
| 53 | child development disorders, pervasive/ |
| 54 | autis*.ti,ab. |
| 55 | f?etal alcohol syndrome.ti,ab. |
| 56 | fragile x syndrome.ti,ab. |
| 57 | down* syndrome.ti,ab. |
| 58 | asperger*.ti,ab. |
| 59 | rett syndrome.ti,ab. |
| 60 | heller* syndrome.ti,ab. |
| 61 | pervasive development* disorder*.ti,ab. |
| 62 | attention deficit hyperactivity disorder.ti,ab. |
| 63 | ADHD.ti,ab. |
| 64 | neurodivers*.ti,ab. |
| 65 | Communication Disorders/ |
| 66 | (communicat* adj1 (disab* or handicap* or impair* or abnormal* or defect*)).ti,ab. |
| 67 | (speech adj1 (disab* or handicap* or impair* or abnormal* or defect*)).ti,ab. |
| 68 | (language adj1 (disab* or handicap* or impair* or abnormal* or defect*)).ti,ab. |
| 69 | Dysarthria.ti,ab. |
| 70 | 7 or 8 or 9 or 10 or 11 or 12 or 13 or 14 or 15 or 16 or 17 or 18 or 19 or 20 or 21 or 22 or 23 or 24 or 25 or 26 or 27 or 28 or 29 or 30 or 31 or 32 or 33 or 34 or 35 or 36 or 37 or 38 or 39 or 40 or 41 or 42 or 43 or 44 or 45 or 46 or 47 or 48 or 49 or 50 or 51 or 52 or 53 or 54 or 55 or 56 or 57 or 58 or 59 or 60 or 61 or 62 or 63 or 64 or 65 or 66 or 67 or 68 or 69 |
| 71 | (child* or youth or adolescent* or girl or boy or teen* or juvenile* or infant* or baby* or toddler* or minor).mp. [mp=title, abstract, heading word, drug trade name, original title, device manufacturer, drug manufacturer, device trade name, keyword heading word, floating subheading word, candidate term word] |
| 72 | 70 and 71 |
| 73 | 6 or 72 |
| 74 | exp Mortality/ |
| 75 | death/ |
| 76 | Death Certificate/ |
| 77 | Mortality.tw. |
| 78 | Death$.tw. |
| 79 | (fatality or fatalities).tw. |
| 80 | (autopsy or coroner report).tw. |
| 81 | 74 or 75 or 76 or 77 or 78 or 79 or 80 |
| 82 | cohort analysis/ |
| 83 | longitudinal study/ |
| 84 | cross-sectional study/ |
| 85 | questionnaire/ |
| 86 | health survey/ or health care survey/ |
| 87 | case control/ |
| 88 | 82 or 83 or 84 or 85 or 86 or 87 |
| 89 | 73 and 81 and 88 |
|  | limit to >yr2000 and 'human' |

Note: Our original search strategy did not include Global Index Medicus, which may index regional literature not captured in major bibliographic databases. However, supplementary searches of Global Index Medicus undertaken during peer review identified no additional eligible studies.

## Table B: Excluded full texts and reasons for exclusion(n=53)

| Author Name /Year | Title | Reason For Exclusion |
| --- | --- | --- |
| Aggarwal (2026) | Pediatric Status Asthmaticus Admissions in Patients With Down Syndrome: National Estimates, United States, 2016-2022 | Mortality data not disaggregated by age for children and adolescents |
| Akhmedullin (2024) | Epilepsy trends in Kazakhstan: A retrospective longitudinal study using data from unified national electronic health system 2014-2020 | Comparison group was within the disability group |
| Autry (2010) | Increased risk of death among children with Lennox-Gastaut syndrome and infantile spasms. | Mortality data not disaggregated by age |
| Bell (2008) | Drowning in people with epilepsy: how great is the risk? | Patient population was people with epilepsy |
| Bilder (2013) | Excess mortality and causes of death in autism spectrum disorders: A follow up of the 1980s Utah/UCLA autism epidemiologic study | Mortality data not disaggregated by age for children and adolescents |
| Blair (2019) | Survival and mortality in cerebral palsy: Observations to the sixth decade from a data linkage study of a total population register and National Death Index | Mortality data not disaggregated by age for children and adolescents |
| Blohme (2000) | Visually impaired Swedish children. The 1980 cohort study - aspects on morality | Comparison group was within the disability group |
| Chen (2019) | Attention-Deficit/Hyperactivity Disorder and mortality risk in Taiwan | Mortality data not disaggregated by age for children and adolescents |
| Day (2005) | Causes of death in remote symptomatic epilepsy. | Mortality data not disaggregated by age for children and adolescents |
| Florio (2015) | Mortality among a cohort of persons with an intellectual disability in New South Wales, Australia | Mortality data not disaggregated by age for children and adolescents |
| Folkestad (2017) | Mortality and causes of death in patients with osteogenesis imperfecta. A register-based national cohort study | Conference abstract with fewer results |
| Folkestad (2018) | Mortality and morbidity in patients with osteogenesis imperfecta in Denmark. | Thesis with fewer results on mortality |
| Gachter (2023) | Down Syndrome: Lifetime burden of disease on nationwide hospitalization data | Abstract with insufficient results |
| Hermon (2001) | Mortality and cancer incidence in persons with Down's syndrome, their parents and siblings | Mortality data is not presented |
| Hill (2003) | Mortality and cancer incidence among individuals with Down syndrome | Mortality data not disaggregated by age for children and adolescents |
| Holst (2013) | Epilepsy and risk of death and sudden unexpected death in the young: A nationwide study | Mortality data not disaggregated by age for children and adolescents |
| Hughes-McCormack (2019) | Comparing mortality and hospital admissions to five years of age in children with and without down syndrome within and across counties 2003-2012: An electronic record matched cohort study in Scotland and Wales | Mortality data not disaggregated by age for children and adolescents |
| Hughes-McCormack (2023) | Rates, causes and predictors of mortality and avoidable mortality in children and young people with and without intellectual disabilities | Results are the same as 2022 paper |
| Hwang (2019) | Mortality and cause of death of Australians on the autism spectrum | Mortality data is not presented |
| Jahan (2019) | Mortality in children with cerebral palsy in rural Bangladesh: a population-based surveillance study | Comparison was within children with disability |
| Janoff (2023) | Incidence and clinical outcomes of pneumonia in persons with down syndrome in the United States | Adult population |
| Jeng (2025) | Longitudinal study on all-cause and suicide mortality among individuals with attention deficit hyperactivity disorder | Mortality data not disaggregated by age for children and adolescents |
| Jokiranta-Olkoniemi (2021) | Risk for Premature Mortality and Intentional Self-harm in autism spectrum disorders | Adult population |
| Kennedy 2026 | Risk of hospitalization and death among autistic young people in England during the Covid-19 pandemic | Participants had underlying conditions |
| Kerac (2012) | Impact of disability on survival from severe acute malnutrition in a developing country setting-a longitudinal cohort study | All participants had malnutrition |
| Kiejna (2023) | Mortality in people with mental disorders in Poland: A nationwide, register-based cohort study | Mortality data not disaggregated by age for children and adolescents |
| Kirby (2019) | A 20-year study of suicide death in a statewide autism population | Mortality data not disaggregated by age for children and adolescents |
| Lee (2017) | Impact of disability status on suicide risks in South Korea: Analysis of National Health Insurance cohort data from 2003 to 2013. | Adult population |
| Lee (2020) | Incidence, comorbidity, and mortality of primary congenital glaucoma in Korea from 2001 to 2015: a nationwide population-based study | Mortality data is not presented |
| Lelijveld (2020) | Long-term outcomes for children with disability and severe acute malnutrition in Malawi. | All study participants had malnutrition |
| Liao (2022) | Mortality rate, risk factors, and causes of death in people with epilepsy and intellectual disability | Comparison was within the disability group |
| Meier (2017) | Mortality risk in a nationwide cohort of individuals with tic disorders and with tourette syndrome | Mortality data not disaggregated by age for children and adolescents |
| Nam (2026) | Adolescent learning disability and premature mortality: A 30-year prospective study of gender differences | Mortality data not disaggregated by age for children and adolescents |
| Ngugi (2014) | Premature mortality in active convulsive epilepsy in rural Kenya: Causes and associated factors | Participants had an underlying condition |
| O'grady (2020) | Survival of individuals with cerebral palsy born in Victoria, Australia, between 1970 and 2011 | Conference abstract with insufficient results |
| Olusanya (2019) | Premature mortality in children with developmental disabilities. | Commentary |
| Ozalp (2025) | The Impact of Down syndrome on perioperative anaesthetic management and outcomes in Infants undergoing isolated ventricular septal defect closure | Mortality data not presented |
| Philippoteaux (2025) | Mortality and fracture risk in children with osteogenesis imperfecta: results from the French nationwide hospital discharge database | Preprint – not peer reviewed |
| Reid (2012) | Survival of individuals with cerebral palsy born in Victoria, Australia, between 1970 and 2004 | Mortality data not disaggregated by age for children and adolescents |
| Reppermund (2019) | Health and wellbeing of people with intellectual disability in New South Wales, Australia: A data linkage cohort | Comparison was within the disability group |
| Richmond-Rakerd (2021) | Longitudinal Associations of Mental Disorders with Physical Diseases and Mortality among 2.3 Million New Zealand Citizens | Mortality data not disaggregated by age for children and adolescents |
| Sarno (2020) | Significant Improvements in Mortality After the Fontan Operation in Children With Down Syndrome | Participants included children with heterotaxy syndrome |
| Schendel (2016) | Association of psychiatric and neurologic comorbidity with mortality among persons with autism spectrum disorder in a Danish population | Mortality data not disaggregated by age for children and adolescents |
| Schmidt (2021) | Outcomes of patients with Down syndrome and acute leukemia: A retrospective observational study | Mortality data is not presented |
| Shaw (2026) | Mortality Among Youth and Young Adults With Autism Spectrum Disorder, Intellectual Disability, or Cerebral Palsy | Mortality data not disaggregated by age for children and adolescents |
| Smith (2019) | Comparing mortality of children and young people with and without intellectual disabilities in Scotland’s annual pupil census: A record linkage study | Mortality data is not presented |
| Srasuebkul 2026 | Life expectancy of people with intellectual disability: a retrospective cohort study from New South Wales, Australia | No comparator data |
| Strafford (2024) | Health care utilization and mortality for people with epilepsy during COVID-19: A population study | Adult population |
| Tann (2018) | Early Childhood Outcomes After Neonatal Encephalopathy in Uganda: A Cohort Study | Mortality data is not presented |
| Vu (2024) | Mortality risk among Autistic children and young people: A nationwide birth cohort study | Comparison group was the young adult population |
| Weijerman (2008) | Prevalence, neonatal characteristics, and first-year mortality of Down syndrome: a national study. | Study population included premature children |
| Yim (2018) | Comparison of growth and mortality between general population and cerebral palsy: A nationwide birth cohort with 10-year follow-up | Comparison by birthweight |
| Zhang (2022) | Mortality in patients with psychogenic non-epileptic seizures a population-based cohort study | Mortality data not disaggregated by age for children and adolescents |

## Table C: JBI Critical appraisal for cohort studies

| **Study Author (Year)** | **1. Were the two groups similar and recruited from the same population** | **2. Were the exposures measured similarly to assign people to both exposed and unexposed groups?** | **3. Was the exposure measured in a valid and reliable way?** | **4.Were confounding factors identified?** | **5. Were strategies to deal with confounding factors stated?** | **6.Were the groups/participants free of the outcome at the start of the study (or at the moment of exposure)?** | **7. Were the outcomes measured in a valid and reliable way?** | **8. Was the follow up time reported and sufficient to be long enough for outcomes to occur?** | **9. Was follow up complete, and if not, were** the  **reasons to loss to follow up described and explored?** | **10. Were strategies to address incomplete follow up utilized?** | **11. Was appropriate statistical analysis used?** | **Overall Score** | **Risk of Bias** |
| --- | --- | --- | --- | --- | --- | --- | --- | --- | --- | --- | --- | --- | --- |
| Abuga (2019) | Y | Y | Y | Y | Y | Y | Y | Y | Y | Y | Y | 100 | L |
| Bourke (2017) | Y | Y | Y | Y | Y | Y | Y | Y | Y | Y | Y | 100 | L |
| Bowden (2026) | Y | Y | P | Y | Y | Y | Y | Y | Y | Y | Y | 91 | L |
| Cardim (2025) | Y | Y | Y | Y | Y | Y | Y | Y | Y | Y | Y | 100 | L |
| Dalsgaard (2015) | Y | Y | Y | Y | Y | Y | Y | Y | Y | Y | Y | 100 | L |
| Decoufle (2002) | Y | Y | Y | Y | Y | Y | Y | Y | Y | P | Y | 91 | L |
| Fitzgerald (2019) | Y | Y | Y | Y | Y | Y | Y | Y | Y | Y | Y | 100 | L |
| Folkestad (2016) | Y | Y | Y | Y | Y | Y | Y | Y | Y | Y | Y | 100 | L |
| Gimeno (2026) | Y | Y | Y | Y | Y | Y | Y | Y | Y | Y | Y | 100 | L |
| Hinwood (2023) | Y | Y | Y | Y | Y | Y | Y | Y | Y | Y | Y | 100 | L |
| Hashmi (2018) | Y | Y | Y | Y | Y | Y | Y | Y | Y | P | Y | 91 | L |
| Huang (2021) | Y | Y | Y | Y | Y | Y | Y | Y | Y | Y | Y | 100 | L |
| Hughes-McCormack (2022) | Y | Y | Y | Y | Y | Y | Y | Y | Y | Y | Y | 100 | L |
| Hughes-McCormack (2020) | Y | Y | Y | Y | Y | Y | Y | Y | Y | Y | Y | 100 | L |
| Kim (2019) | Y | Y | Y | Y | Y | Y | Y | Y | Y | Y | Y | 100 | L |
| Kim (2021) | Y | Y | Y | Y | Y | Y | Y | Y | Y | Y | Y | 100 | L |
| Macaulay (2026) | Y | Y | Y | Y | N | Y | Y | Y | Y | Y | Y | 91 | L |
| McCarron (2015) | Y | Y | Y | Y | Y | Y | Y | Y | Y | Y | Y | 100 | L |
| Namaganda (2020) | Y | Y | Y | Y | Y | Y | Y | Y | Y | Y | Y | 100 | L |
| McCarthy (2009) | Y | Y | Y | Y | Y | Y | Y | Y | Y | Y | Y | 100 | L |
| Patja (2001) | Y | Y | Y | Y | Y | Y | Y | Y | Y | Y | Y | 100 | L |
| Sherrard (2001) | Y | Y | Y | Y | Y | Y | Y | Y | P | P | Y | 82 | M |
| Shumway (2025) | Y | Y | Y | Y | Y | Y | Y | Y | Y | Y | Y | 100 | L |
| Smith (2020) | Y | Y | Y | Y | Y | Y | Y | Y | Y | Y | Y | 100 | L |
| Smith (2021) | Y | Y | Y | Y | Y | Y | Y | Y | Y | Y | Y | 100 | L |
| Sun (2019) | Y | Y | Y | Y | Y | Y | Y | Y | Y | Y | Y | 100 | L |
| Tibby (2012) | Y | Y | Y | Y | Y | Y | Y | Y | Y | Y | Y | 100 | L |
| Yoo (2022) | Y | Y | Y | Y | Y | Y | Y | Y | Y | Y | Y | 100 | L |
| Zylbersztejn (2025) | Y | Y | Y | P | P | Y | Y | Y | Y | Y | Y | 82 | M |

*Y: Yes, P: Partially, M: Medium, L: low,

## Table D: Disability definitions as reported by authors of individual studies (n=29 studies)

| **Author name** | **Title** | **Disability Definition** |
| --- | --- | --- |
| Abuga (2019) | Premature mortality in children aged 6–9 years with  neurological impairments in rural Kenya: a cohort study | Neurological impairment |
| Bourke (2017) | Twenty-Five Year Survival of Children with Intellectual Disability in Western Australia | Intellectual disability |
| Bowden (2026) | Mortality Risk of Youth With Neurodevelopmental Conditions: An Aotearoa New Zealand Nationwide Birth Cohort Study | Attention-deficit/  hyperactivity disorder (ADHD), autism spectrum disorder  (hereafter, autism), fetal alcohol spectrum disorder (FASD),intellectual  disability (ID), communication and language disorders,  and specific learning and motor disorders |
| Cardim (2025) | All-Cause and Cause-Specific Mortality in Children with Congenital Zika Syndrome in Brazil | Congenital zika syndrome |
| Dalsgaard (2015) | Mortality in children, adolescents, and adults with attention deficit hyperactivity disorder: a nationwide cohort study | Attention-Deficit Hyperactivity Disorder |
| Decoufle (2002) | Increased mortality in children and adolescents with developmental disabilities | Intellectual disability, Hearing impairment, Visual impairment & Cerebral palsy |
| Fitzgerald (2019) | Suicidal behaviour among persons with attention-deficit hyperactivity disorder | Attention-Deficit Hyperactivity Disorder |
| Folkestad (2016) | Mortality and morbidity in patients with osteogenesis imperfecta in Denmark | Osteogenesis Imperfecta |
| Gimeno (2026) | Hospital admissions and school absences of primary school children with and without neurodisability | Neurological impairment |
| Hashmi (2018) | Multicenter study of mortality in achondroplasia | Achondroplasia |
| Hinwood (2023) | Survival of individuals with cerebral palsy in Victoria, Australia: A longitudinal cohort study spanning four decades | Cerebral Palsy |
| Huang (2021) | Adverse outcomes after major surgery in children with intellectual disability | Intellectual disability |
| Hughes-McCormack (2020) | Birth incidence, deaths and hospitalisations of children and young people with down syndrome, 1990-2015: Birth cohort study | Down syndrome |
| Hughes-McCormack (2022) | Rates, causes and predictors of all-cause and avoidable mortality in 163 686 children and young people with and without intellectual disabilities: a record linkage national cohort study | Intellectual disability |
| Kim (2019) | Which growth parameters can affect mortality in cerebral palsy? | Cerebral Palsy |
| Kim (2021) | Mortality of children with autism spectrum disorder using data from a large-scale Korean national cohort | Autism Spectrum Disorder |
| Macaulay (2026) | Mortality Risk Between Ages 11 and 22 Years Among Young People With Neurodisability in England: A National Cohort Study Using Linked Health and Education Data | Neurological impairment |
| McCarron (2015) | Mortality Rates in the General Irish Population Compared to those with an Intellectual Disability from 2003 to 2012 | Intellectual disability |
| McCarthy (2009) | Mortality associated with attention-deficit hyperactivity disorder (ADHD) drug treatment: a retrospective cohort study of children, adolescents and young adults using the general practice research database | Attention-Deficit Hyperactivity Disorder |
| Namaganda (2020) | Excessive premature mortality among children with cerebral palsy in rural Uganda: A longitudinal, population-based study | Cerebral Palsy |
| Patja (2001) | Cause-specific mortality of people with intellectual disability in a population-based, 35-year follow-up study | Intellectual disability |
| Sherrard (2001) | Injury in young people with intellectual disability: descriptive epidemiology | Intellectual disability |
| Shumway (2025) | Hospital-recorded chronic health conditions in children with and without Down syndrome in England: a national cohort of births from 2003 to 2019 | Down syndrome |
| Smith (2020) | Rates and causes of mortality among children and young people with and without intellectual disabilities in Scotland: a record linkage cohort study of 796 190 school children | Intellectual disability |
| Smith (2021) | Mortality in 787,666 school pupils with and without autism: A cohort study | Autism Spectrum Disorder |
| Sun (2019) | Association of psychiatric comorbidity with the risk of premature death among children and adults with attention-deficit/hyperactivity disorder | Attention-Deficit Hyperactivity Disorder |
| Tibby (2012) | Clinical course and outcome for critically ill children with Down syndrome: A retrospective cohort study | Down syndrome |
| Yoo (2022) | Prevalence and Premature Mortality Statistics of Autism Spectrum Disorder Among Children in Korea: A Nationwide Population-Based Birth Cohort Study | Autism Spectrum Disorder |
| Zylbersztejn (2025) | Phenotyping Neurodisability in Hospital Records  in England: A National Birth Cohort Using Linked  Administrative Data | Neurological impairment |

## Table E: Meta-analysis tables – Overall results (n=26 comparison groups)

| Author (year) | Effect size (95% CIs) |
| --- | --- |
| Abuga (2019) | 3.15(1.66,5.49) |
| Bourke (2017)^a^ | 6.0(4.8,7.6) |
| Bourke (2017)^b^ | 12.6(9.0,17.7) |
| Bowden (2026) | 4.83(3.43, 6.00) |
| Cardim (2025) | 13.1(11.86,14.5) |
| Dalsgaard (2015)^a^ | 1.97(0.99,3.46) |
| Dalsgaard (2015)^b^ | 1.63(1.25,2.09) |
| Folkestad (2016) | 68.1(16.2,287.3) |
| Hashmi (2018)^a^ | 0.14(0.02,0.98) |
| Hashmi (2018)^b^ | 2.40(1.00, 5.76) |
| Hashmi (2018)^c^ | 0.30(0.08,1.20) |
| Huang (2020) | 2.04(1.05,3.93) |
| Hughes-McCormack (2022)^a^ | 27.4(20.6,36.3) |
| Hughes-McCormack (2022)^b^ | 15.8(12.7,19.8) |
| Hughes-McCormack (2022)^c^ | 8.6(6.9,10.7) |
| Kim (2019)^a^ | 11.64(6.32,21.44) |
| Kim (2019)^b^ | 11.27(5.92,21.46) |
| Kim (2019)^c^ | 15.38(8.44,28.02) |
| Kim (2019)^d^ | 18.03(9.13,35.62) |
| Kim (2019)^e^ | 24.85(9.97,61.91) |
| Kim (2021) | 2.5(2.2,2.9) |
| McCarron (2015) | 6.68(5.91,7.52) |
| Smith (2020)^a^ | 21.6(16.6,28.2) |
| Smith (2021) | 1.1(0.5,2.5) |
| Sun (2019) | 1.41(0.97,2.04) |
| Tibby (2012) | 0.83(0.63,1.09) |

## Table F: Results by age group (0 – 5 years) (n =10 comparison groups)

| Author (Year) | Effect size (95% CIs) |
| --- | --- |
| Bourke (2017)^a^ | 6.0(4.8,7.6) |
| Dalsgaard (2015)^a^ | 1.97(0.99,3.46) |
| Kim (2019)^a^ | 11.64(6.32,21.44) |
| Kim (2019)^b^ | 11.27(5.92,21.46) |
| Kim (2019)^c^ | 15.38(8.44,28.02) |
| Kim (2019)^d^ | 18.03(9.13,35.62) |
| Kim (2019)^e^ | 24.85(9.97,61.91) |
| Cardim (2025) | 13.1(11.86,14.5) |
| Hashmi (2018)^a^ | 0.14(0.02,0.98) |
| Hashmi (2018)^b^ | 2.40(1.00,5.76) |

## Table G: Results by age group (6 – 19 years) (n =7 comparison groups)

| Author (Year) | Effect size (95% CIs) |
| --- | --- |
| Abuga (2019) | 3.15(1.66,5.49) |
| Bourke (2017)^b^ | 12.6(9.0,17.7) |
| Dalsgaard (2015)^b^ | 1.63(1.25,2.09) |
| Huang (2021) | 2.04(1.05,3.93) |
| Hughes-McCormack (2022)^b^ | 15.8(12.7,19.8) |
| Hughes-McCormack (2022)^c^ | 8.6(6.9,10.7) |
| Kim (2021) | 2.5(2.2,2.9) |

## Table H: Intellectual and developmental disability (n =14 comparison groups)

| Author (year) | Effect size (95% CIs) |
| --- | --- |
| Bourke (2017)^a^ | 6.0(4.8,7.6) |
| Bourke (2017)^b^ | 12.6(9.0,17.7) |
| Dalsgaard (2015)^a^ | 1.97(0.99,3.46) |
| Dalsgaard (2015)^b^ | 1.63(1.25,2.09) |
| Huang (2021) | 2.04(1.05,3.93) |
| Hughes-McCormack (2022)^a^ | 27.4(20.6,36.3) |
| Hughes-McCormack (2022)^b^ | 15.8(12.7,19.8) |
| Hughes-McCormack (2022)^c^ | 8.6(6.9,10.7) |
| Kim (2021) | 2.5(2.2,2.9) |
| McCarron (2015) | 6.68(5.91,7.52) |
| Smith (2020) | 21.6(16.6,28.2) |
| Smith (2021) | 1.1(0.5,2.5) |
| Sun (2019) | 1.41(0.97,2.04) |
| Tibby (2012) | 0.83(0.63,1.09) |

## Table I: Neurological disability (n = 7 comparison groups)

| Author (year) | Effect size (95% CIs) |
| --- | --- |
| Abuga (2019) | 3.15(1.66,5.49) |
| Kim (2019)^a^ | 11.64(6.32,21.44) |
| Kim (2019)^b^ | 11.27(5.92,21.46) |
| Kim (2019)^c^ | 15.38(8.44,28.02) |
| Kim (2019)^d^ | 18.03(9.13,35.62) |
| Kim (2019)^e^ | 24.85(9.97,61.91) |
| Cardim (2025) | 13.1(11.86,14.5) |

## Table J: Physical disability (n = 4 comparison groups)

| Author (year) | Effect size (95% CIs) |
| --- | --- |
| Folkestad (2016) | 68.1(16.2,287.3) |
| Hashmi (2018)^a^ | 0.14 (0.02,0.98) |
| Hashmi (2018)^b^ | 2.40 (1.00 ,5.76) |
| Hashmi (2018)^c^ | 0.30 (0.08 ,1.20) |

## Fig A: Sensitivity analysis for the overall association of disability and all-cause mortality among children and adolescents, restricted to one estimate per study

## Fig B: Sensitivity analysis for the association of disability and all-cause mortality among children aged 0 - 5 years and 6-19 years, restricted to one estimate per study

## Fig C: Sensitivity analysis for the association of disability type with all-cause mortality for children and adolescents, restricted to one estimate per study

# Fig D: Sensitivity analysis for the overall association of disability and mortality with only minimally adjusted effect sizes

## Fig E: Sensitivity analysis for the overall association of disability and mortality with only multivariable adjusted effect sizes

# Fig F: Funnel plot assessing publication bias in studies examining the association between disability and all-cause mortality among children and adolescents
